# Role of C-Reactive Protein at Sites of Inflammation and Infection

**DOI:** 10.3389/fimmu.2018.00754

**Published:** 2018-04-13

**Authors:** Nicola R. Sproston, Jason J. Ashworth

**Affiliations:** School of Healthcare Science, Manchester Metropolitan University, Manchester, United Kingdom

**Keywords:** C-reactive protein, native C-reactive protein, monomeric C-reactive protein, inflammation, infection

## Abstract

C-reactive protein (CRP) is an acute inflammatory protein that increases up to 1,000-fold at sites of infection or inflammation. CRP is produced as a homopentameric protein, termed native CRP (nCRP), which can irreversibly dissociate at sites of inflammation and infection into five separate monomers, termed monomeric CRP (mCRP). CRP is synthesized primarily in liver hepatocytes but also by smooth muscle cells, macrophages, endothelial cells, lymphocytes, and adipocytes. Evidence suggests that estrogen in the form of hormone replacement therapy influences CRP levels in the elderly. Having been traditionally utilized as a marker of infection and cardiovascular events, there is now growing evidence that CRP plays important roles in inflammatory processes and host responses to infection including the complement pathway, apoptosis, phagocytosis, nitric oxide (NO) release, and the production of cytokines, particularly interleukin-6 and tumor necrosis factor-α. Unlike more recent publications, the findings of early work on CRP can seem somewhat unclear and at times conflicting since it was often not specified which particular CRP isoform was measured or utilized in experiments and whether responses attributed to nCRP were in fact possibly due to dissociation into mCRP or lipopolysaccharide contamination. In addition, since antibodies for mCRP are not commercially available, few laboratories are able to conduct studies investigating the mCRP isoform. Despite these issues and the fact that most CRP research to date has focused on vascular disorders, there is mounting evidence that CRP isoforms have distinct biological properties, with nCRP often exhibiting more anti-inflammatory activities compared to mCRP. The nCRP isoform activates the classical complement pathway, induces phagocytosis, and promotes apoptosis. On the other hand, mCRP promotes the chemotaxis and recruitment of circulating leukocytes to areas of inflammation and can delay apoptosis. The nCRP and mCRP isoforms work in opposing directions to inhibit and induce NO production, respectively. In terms of pro-inflammatory cytokine production, mCRP increases interleukin-8 and monocyte chemoattractant protein-1 production, whereas nCRP has no detectable effect on their levels. Further studies are needed to expand on these emerging findings and to fully characterize the differential roles that each CRP isoform plays at sites of local inflammation and infection.

## C-Reactive Protein (CRP)

C-reactive protein is a homopentameric acute-phase inflammatory protein, a highly conserved plasma protein that was initially discovered in 1930 by Tillet and Francis while investigating the sera of patients suffering from the acute stage of *Pneumococcus* infection and was named for its reaction with the capsular (C)-polysaccharide of *Pneumococcus* (1). In the presence of calcium, CRP binds to polysaccharides such as phosphocholine (PCh) on microorganisms and triggers the classical complement pathway of innate immunity by activating C1q (2). CRP has many homologs in vertebrates and some invertebrates (3) and is a member of the pentraxin family, which includes other structurally related molecules such as serum amyloid A (4). Transcriptional induction of the *CRP* gene mainly occurs in hepatocytes in the liver in response to increased levels of inflammatory cytokines, especially interleukin-6 (IL-6) (5).

C-reactive protein exhibits elevated expression during inflammatory conditions such as rheumatoid arthritis, some cardiovascular diseases, and infection (6). As an acute-phase protein, the plasma concentration of CRP deviates by at least 25% during inflammatory disorders (7). The highest concentrations of CRP are found in serum, with some bacterial infections increasing levels up to 1,000-fold (8). However, when the stimuli ends, CRP values decrease exponentially over 18–20 h, close to the half-life of CRP (9). CRP plasma levels increase from around 1 µg/mL to over 500 µg/mL within 24–72 h of severe tissue damage such as trauma and progressive cancer (10). IL-6 is reported to be the main inducer of *CRP* gene expression, with IL-1 enhancing the effect (11). However, although IL-6 is necessary for *CRP* gene induction, it is not sufficient to achieve this alone (12).

There are many factors that can alter baseline CRP levels including age, gender, smoking status, weight, lipid levels, and blood pressure (13). The average levels of CRP in serum in a healthy Caucasian is around 0.8 mg/L, but this baseline can vary greatly in individuals due to other factors, including polymorphisms in the *CRP* gene (14). The human *CRP* gene can be found at 1q23.2 on the long arm of chromosome 1, and to date, there have been no allelic variations or genetic deficiencies discovered for this gene although some polymorphisms have been identified (13). For example, up to 50% of baseline variance in CRP is associated with the number of dinucleotide repeats found in an intronic region of the gene (15).

There is no significant seasonal variation in baseline CRP concentration; however, twin studies show a significant heritable component in baseline CRP values that is independent of age and body mass index (16). Pankow et al. (17) found evidence that interindividual variation in blood CRP levels is 35–40% heritable. Increased CRP levels are typically associated with disease, but liver failure is one condition observed to impair CRP production. Very few drugs reduce elevated CRP levels unless they treat the underlying pathology that is causing the acute-phase stimulus (16).

There is emerging research that oral hormone replacement therapy (HRT) causes background levels of circulating CRP to increase in postmenopausal women, increasing the risk of thrombotic events such as clots (18). Corcoran et al. (19) found that a combination of estrogen and oxidized low-density lipoproteins (oxLDLs) increased CRP expression in a model of coronary heart disease in both older men and postmenopausal women, but no effect on CRP expression was seen when estrogen supplementation was replaced with testosterone. Ridker et al. (20) found that healthy postmenopausal women had nearly twofold increased levels of circulating CRP when they were taking oral HRT and that CRP was the most affected inflammatory marker. Numerous studies have confirmed that CRP is a predictive marker for cardiovascular disease and that HRT use in postmenopausal women increases the risk of stroke and blood clots (20–23).

Interestingly the mode of HRT delivery appears to influence the effect on circulating CRP levels. Vongpatanasin et al. (23) found that estrogen administered orally increases circulating CRP levels twofold, whereas estrogen administered transdermally had no effect on circulating CRP levels. Similarly, patients taking oral HRT containing estrogens combined with progestogens had an increase in circulating CRP levels in the first 12 months of therapy compared to those using transdermal therapy who demonstrated no change in circulating CRP levels (22). In contrast, several other studies have instead shown that circulating CRP levels are reduced in humans treated with transdermal estrogen (24, 25). A reduction in CRP levels following peripheral estrogen administration supports the findings of Ashcroft et al. (26) demonstrating that estrogen reduces the inflammatory response during wound healing. The effect of transdermal administration of estrogen on local CRP levels in peripheral tissues such as skin has not yet been elucidated, with previous studies measuring only circulating levels of CRP.

## Isoforms of CRP

The pentameric protein, termed native CRP (nCRP), is characterized by a discoid configuration of five identical non-covalently bound subunits, each 206 amino acids long with a molecular mass of about 23 kDa. These five subunits lie in the same orientation around a central pore and arranged in a characteristic “lectin fold” with a two-layered beta sheet (15). Each subunit lies with the PCh binding site facing the “recognition” face of the nCRP molecule (27). The molecule has a ligand-binding face that has a characteristic feature of having two calcium ions per protomer. The calcium ions are important for the stability and binding of ligands. The “opposite” face interacts with the C1q aspect of the complement pathway as well as interacting with Fc receptors (6).

The pentameric protein is synthesized primarily in liver hepatocytes but has also been reported to be synthesized in other cell types such as smooth muscle cells (28), macrophages (29), endothelial cells (30), lymphocytes, and adipocytes (31). CRP is first synthesized as monomers and then assembled into the pentamer in the endoplasmic reticulum of the source cell. In hepatocytes, the pentameric protein is retained in the endoplasmic reticulum by binding to two carboxylesterases, gp60a and gp50b (32). While in a resting (non-inflammatory) state, CRP is released slowly from the endoplasmic reticulum, but following an increase in inflammatory cytokine levels, the binding CRP to the carboxylesterases decreases and CRP is secreted rapidly (6). The stimulation of CRP synthesis mainly occurs in response to pro-inflammatory cytokines, most notably IL-6 and to a lesser degree IL-1 and tumor necrosis alpha (TNF-α) (33).

Pentameric CRP can be irreversibly dissociated, with the resultant free subunits termed monomeric (or modified) CRP (mCRP). The dissociation of nCRP into free subunits has been observed at either high concentrations of urea (34) or high temperatures in the absence of calcium (35). The mCRP molecules are distinguished from nCRP by their different antigenic, biological, and electrophoretic activities (36) and by the fact that they express different neoepitopes (37). The two isoforms of CRP have been shown to have distinct biological functions in the inflammatory process. For example, Khreiss et al. (37) provided evidence that nCRP suppresses the adherence of platelets to neutrophils, whereas mCRP enhances these interactions. This difference in function can be explained by the two isoforms binding to differing types of Fcgamma (Fcγ)-receptor involved in the signaling process. The mCRP isoform utilizes the low-affinity immune complex binding immunoglobulin G (IgG) receptor called FcγRIIIb (CD16b) on neutrophils and FcγRIIIa (CD16a) on monocytes, while nCRP binds to the low-affinity IgG receptor FcγRIIa (CD32) (38).

Evidence is emerging of new structural intermediates of CRP with biological function. Ji et al. (39) found that the native protein first dissociates into subunits while retaining some of the native conformation before fully dissociating into mCRP. This intermediate, termed mCRP_m_, is formed when the nCRP is bound to cell membranes and then dissociates, allowing the subunits to retain some of the conformation before fully dissociating into mCRP subunits on detachment from the membrane. It is suggested that this transitional process allows for more effective regulation of CRP function, with mCRP_m_ allowing for the enhanced activation of the classical complement pathway (39). Further work needs to be conducted to determine the biological functions of the mCRP_m_ intermediate, but initial findings suggest that it behaves in a similar manner to mCRP, typically promoting pro-inflammatory activity.

## CRP in Disease Pathology

The majority of CRP research has focused on the role of CRP and its isoforms on cardiovascular disease and stroke. CRP is used as a clinical marker of inflammation, with elevated serum levels being a strong independent predictor of cardiovascular disease in asymptomatic individuals (40). CRP levels have been linked to prognosis in patients with atherosclerotic disease, congestive heart failure, atrial fibrillation, myocarditis, aortic valve disease, and heart transplantation, suggesting that it has an active role in the pathophysiology of cardiovascular disease (41). High-sensitivity assays, such as nephelometric assays, are used to detect baseline levels of CRP and patients who are at risk of cardiovascular disease. An individual with a CRP level higher than 3 mg/L has an increased risk of coronary heart disease (42), and this risk increases in those with type 2 diabetes (43).

Increased levels of CRP have been found in patients with appendicitis, cholecystitis, pancreatitis, and meningitis (44). In patients suffering possible symptoms of appendicitis, acute appendicitis can be excluded in those with CRP levels lower than 25 mg/L in blood taken 12 h after the onset of symptoms (45). When clinical symptoms of cholecystitis occur concurrently with CRP levels of over 30 mg/L, an accurate diagnosis of cholecystitis can be obtained with 78% sensitivity, suggesting that CRP is a more sensitive marker than erythrocyte sedimentation rate and white cell count in supporting cholecystitis diagnosis (46). In terms of acute pancreatitis, CRP levels of more than 210 mg/L were able to discriminate between mild and severe cases, with 83% sensitivity and 85% specificity (47). Serum CRP is elevated in bacterial meningitis, and resolution of symptoms following treatment with antibiotics is slow in those with the highest CRP levels (48). Measurement of CRP in cerebrospinal fluid has a sensitivity of 100% and a specificity of 94% for differentiating between patients with bacterial meningitis, viral meningitis, and no infection (49).

Although studies have shown that CRP levels increase during infections and inflammatory diseases, the precise role of CRP isoforms in their development and progression remains largely unknown. Thus, urgent investigations are required to determine the effects of each CRP isoform on specific cellular processes during disease development. Evidence shows that in general nCRP tends to exhibit more anti-inflammatory activities relative to the mCRP isoform, possibly because nCRP limits the generation of the membrane attack complex (MAC) and C5a, thus inhibiting the alternative complement activation (50). In contrast, mCRP can have marked pro-inflammatory properties both *in vitro* and *in vivo* by promoting monocyte chemotaxis and the recruitment of circulating leukocytes to areas of inflammation *via* Fcy-RI and Fcy-RIIa signaling (50). Thus, in addition to therapeutic strategies to inhibit CRP activity (51), more targeted therapies have been proposed for the treatment of CRP-mediated pathologies, including inhibiting mCRP activity (52) or preventing the dissociation of nCRP into mCRP (53).

## CRP and Inflammation

**Figure 1:**
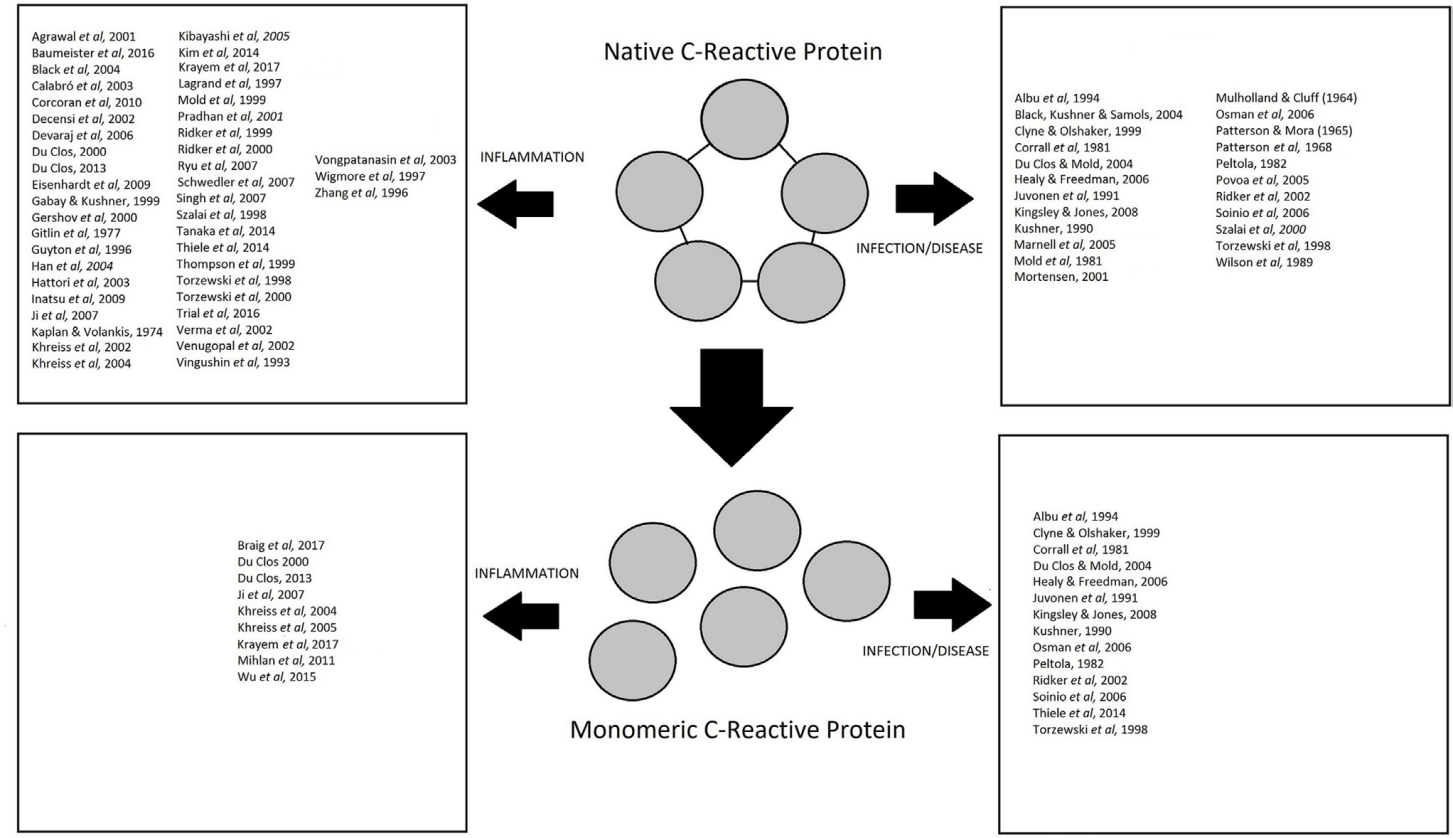
Summary of studies investigating the role of native C-reactive protein (CRP) and monomeric CRP in inflammation, infection, and disease.

C-reactive protein levels are known to increase dramatically in response to injury, infection, and inflammation (Figure 1). CRP is mainly classed as an acute marker of inflammation, but research is starting to indicate important roles that CRP plays in inflammation. CRP is the principal downstream mediator of the acute-phase response following an inflammatory event and is primarily synthesized by IL-6-dependent hepatic biosynthesis (54, 55). The main role of CRP in inflammation tends to focus around the activation of the C1q molecule in the complement pathway leading to the opsonization of pathogens. Although CRP can initiate the fluid phase pathways of the host defense by activating the complement pathway, it can also initiate cell-mediated pathways by activating complement as well as to binding to Fc receptors of IgG (54). CRP binds to Fc receptors with the resulting interaction leading to the release of pro-inflammatory cytokines (56). CRP also has the ability to recognize self and foreign molecules based on the pattern recognition, something that other activators of complement such as IgG cannot achieve because these molecules only recognize distinct antigenic epitopes (56).

Evidence suggests that CRP is not only just a marker of inflammation but also plays an active role in the inflammatory process. However, most early research in the literature only refers to CRP and does not distinguish between the two isoforms. Thus, unlike more recent publications, the findings of early work on CRP can seem somewhat unclear and at times conflicting since it was often not specified which CRP isoform was measured or utilized in experiments, whether responses attributed to nCRP were in fact possibly due to partial/full dissociation into mCRP or if lipopolysaccharide (LPS) contamination could be present. More recent studies generally distinguish between the differential effects of each CRP isoform on inflammatory processes, but since antibodies for mCRP are not commercially available to date, few laboratories are able to conduct studies investigating the mCRP isoform.

There is increasing evidence that CRP has a functional role in the inflammatory process. It is well established that CRP is an acute marker of inflammation and that its concentration increases in circulation during inflammatory events. CRP is deposited at sites of inflammation and tissue damage in both naturally occurring and experimental conditions (57). However, there is a raft of published data investigating CRP that does not consider its two different isoforms. Understandably, when some of these studies were conducted, the existence of two CRP isoforms was not well established and available antibodies would have been raised against the pentameric nCRP alone. Another issue with published data is that CRP localization is often investigated in only a narrow range of inflammatory conditions and tissue types. Although the mCRP isoform has been shown to be insoluble in plasma, it becomes localized in inflamed tissues and amplifies a pro-inflammatory response by a positive feedback loop (58).

The literature suggests that CRP binds to damaged cell membranes and contributes to the inflammatory response (59), with CRP molecules becoming associated with terminal complement complexes, especially in atherosclerotic lesions (60). Lagrand et al. (61) provided evidence that CRP localizes to infarcted heart tissue and promotes local complement activation, triggering further damage to the heart tissue. Gitlin et al. (62) concluded that CRP was localized to the nuclei of cells within the synovium of rheumatoid arthritis patients, but the cell type was not identified at the time. However, other studies indicate no significant localization of CRP in a number of pathologies, suggesting that CRP is found predominantly in the fluid phase rather than becoming deposited in tissues at sites of inflammation or injury (63). There has been little research conducted on the localization of CRP in inflammatory cells to date. There is a correlation between the localization of CRP in neutrophil infiltrates, especially in lesions of vasculitis and allergic encephalomyelitis (64, 65).

## CRP and Infection

C-reactive protein is a marker for inflammation, and its levels increase during bacterial infection (66). Kingsley and Jones (67) stated that CRP increases during infection in response to monocytic mediators such as IL-1 and IL-6 and that it has a stable decay rate. It is thought that most of the interaction between CRP and the immune response to pathogens involves the binding of CRP to PCh and the activation of the classical complement pathway (68). Mold et al. (69) showed that CRP provides mice with protection against infection by the gram-positive pathogen *Streptococcus pneumoniae* by binding to a PCh determinant of the pathogen cell wall and activating the complement pathway. Mice pretreated with 200 µg CRP before being infected showed an increase in percentage survival across all pathogen doses tested. The study concluded that the ability of CRP to protect against infection lies in its ability to bind to pneumococcal polysaccharide C in the bacterial cell wall (69).

Szalai et al. (70) showed that CRP can confer protective benefits against *Salmonella enterica* serovar Typhimurium, a gram-negative pathogen that provides a model of typhoid fever in mice. By using transgenic mice expressing human CRP, the study found that CRP offered protection against a low dose of Typhimurium and increased resistance to a fatal infection with a low dose of Typhimurium. Szalai et al. (70) concluded that CRP increases the early clearance of intravenously injected bacteria from the blood and reduces dissemination of bacteria to the liver and spleen during the initial stages of infection, thus allowing the mice to survive infection.

Marnell et al. (71) reviewed the protective role CRP against *Haemophilus influenza* infection in both transgenic and wild-type mice treated by passive inoculation. CRP was shown to bind the pneumococcal C-polysaccharide of bacteria and opsonize them for phagocytosis. This process did not require the use of the Fcγ receptors, suggesting that CRP is not primarily protective by direct opsonization but more likely through activation of complement and subsequent opsonophagocytosis.

Kingsley and Jones (67) tested whether CRP could be used to distinguish different types of infections. They discovered that mean CRP levels in a spreading infection were higher than those in other colonized, critically colonized, and locally infected groups. All cases of infection showed an increase in CRP levels compared to non-infected controls, but CRP levels could not distinguish between the infection types, showing that it is infection in general that causes CRP levels to increase, rather than the type of infection. This was also noted by Healy and Freedman (66) who showed that CRP levels can be used only as a method of detecting infection, rather than distinguishing it.

C-reactive protein can mediate host responses to *Staphylococcus aureus* including some protective function against infection and an increase in phagocytosis of this pathogen. Povoa et al. (72) stated that the normal CRP level for the healthy population is about 0.08 mg/dL, and this increases to more than 8.7 mg/dL during chronic *S. aureus* infection. Thus, CRP can be used as an indicator of infection, alongside a body temperature of more than 38.2°C. Patterson and Mora (73) observed that enhanced resistance to intraarticular infection with *S. aureus* in chickens was associated with an increase in serum CRP and that isolated preparations of the protein produced antibacterial activity. Mulholland and Cluff (74) discovered that endotoxin-induced changes in resistance to local infection with *S. aureus* in rabbits were correlated with the circulating levels of leukocytes in the blood. The study showed that induced resistance was paralleled by an increase in CRP and leukocytes. This was collaborated by Patterson et al. (75) who found an association between CRP and non-specific resistance to infection, including *S. aureus* and showed that CRP was acting upon the polysaccharide bacterial cell wall. Black et al. (3) stated that CRP enhances the *in vitro* phagocytosis of many microorganisms (including *S. aureus*) by leukocytes. Their work confirmed this finding even in the absence of complement, suggesting that the enhancement of phagocytosis by CRP is due to the interactions with Fcγ receptors.

In summary, evidence shows that CRP is not only a marker of infection and inflammation but that CRP also has a protective role against bacterial infections (Figure 1), principally through the activation of complement and subsequent opsonization of pathogens.

## CRP and Complement

Complement is one of the major defenses of the human immune system that is involved in the clearance of foreign particles and organisms after recognition by antibody. The complement pathway is made up of 35 plasma or membrane proteins that is an important system in immunity and the defense of the host against microbial infection. The components of the complement pathway can be activated in three different pathways to trigger a cascade of proteins, which are used to help bind microbial surfaces for the immune system to recognize and activate phagocytosis (76, 77). The classical pathway is triggered by a target bound antibody, whereas the lectin pathway is triggered by microbial repetitive polysaccharide structures and the alternative pathway is triggered by recognition of other foreign surface structures. Even though the triggers are different, the three pathways merge at a pivotal activation of the C3 and C5 convertases. A majority of the components are synthesized in the liver, C1 in the intestinal epithelium, and factor D in the adipose tissue (76).

The role of CRP in activating the complement pathway has been extensively investigated. In 1974, Kaplan and Volanakis first described the ability of CRP to activate the classical complement pathway using C-polysaccharide and phospholipid ligands (59). The activation of complement by CRP is considered a crucial step since when complement was depleted, and the effects of CRP were abrogated (50).

The opposite face of the CRP molecule, which is typically complexed with polyvalent ligand or chemically cross-linked, binds to C1q and activates the classical complement pathway (56). C1q is a large 460-kDa molecule made up of six identical subunits, each made up of three structurally similar but distinct polypeptide chains (78). This process requires the use of calcium ions for the stable formation of the C1 complex (79). CRP is most effective during the early classical pathway activation of C1, C4, and C2 (80). This is because the ligand-bound interaction with C1q leads to the formation of C3 convertase, triggering the complement activation of C1–C4 but with little activation of the late complement proteins C5–C9 (15).

Activation of complement by CRP varies from activation by antibody in that CRP has selective activation of early components without the need to form the MAC. In addition to activating the classical complement pathway, CRP can inhibit the alternative complement pathway by decreasing C3 and C5 convertase activities and inhibiting the complement amplification loop. This is achieved by the recruitment of factor H to the cell surface and by preventing C5 convertase cleaving C5 to recruit neutrophils and prevent the formation of the MAC (71). As the levels of CRP increase, this causes decreased binding of C3b and C5b-9 to liposomes, possibly also explaining the lack of C5–C9 consumption by CRP during classical pathway activation (80).

Both the initiator (C1q) and the inhibitor (C4bp) of the classic complement pathway compete for mCRP binding, with the competition controlling the local balance of activation and inhibition of the pathway in tissues (58). Interestingly, mCRP but not nCRP binds the C4bp inhibitor, suggesting that mCRP rather than nCRP is able to provide a high degree of control over the classic complement pathway (58).

## CRP and Apoptosis

There has been little research conducted into the effect of CRP on the proliferation process. However, there is evidence that CRP has a major role in the apoptosis process. Devaraj et al. (81) showed that CRP stimulates the production of pro-apoptotic cytokines and inflammatory mediators *via* the activation of Fc-γ receptors. The pro-apoptotic cytokines and inflammatory mediators induced by CRP include interleukin-1β (IL-1β), tumor necrosis factor-α (TNFα), and reactive oxygen species (82, 83).

C-reactive protein induces the upregulation of p53 in monocytes and affects cell cycle kinetics of monocytes through CD32 (FcγRII), inducing apoptosis by G_2_/M arrest in the cell cycle (84). CD32 receptors have been shown to trigger apoptotic signals and are expressed in a subset of monocytes that polarize to pro-inflammatory macrophages, suggesting that CRP may dampen macrophage-driven pro-inflammatory responses by inducing apoptosis (85).

C-reactive protein is elevated in cardiovascular disorders and is a mediator of atherosclerosis. CRP localizes directly in the atherosclerotic plaques where it induces the expression of genes that are directly involved in the adhesion of monocytes and the recruitment of intracellular molecules such as E-selectin and monocyte chemoattractant protein-1 (MCP-1). CRP has also been shown to play a role in mediating low-density lipoprotein uptake in macrophages and activating the complement system, which is implicated in atherogenesis (86). Apoptosis occurs in atherosclerotic plaques and the number of apoptotic cells increase as lesions become more advanced. As cells become apoptotic, they start to cause plaque disruption, leading to the expression of growth arrest- and DNA damage-inducible gene 153 (*GADD153*). *GADD153* upregulation has been shown to induce G_1_ arrest or apoptosis in some cancer cell lines (87). Blaschke et al. (88) found that CRP can induce the apoptosis of human coronary vascular smooth muscle cells through a caspase-mediated mechanism, especially through increased caspase-3 activity. CRP was co-localized to the *GADD153* gene product in atherosclerotic lesions suggesting that CRP is triggering the caspase cascade and apoptosis by inducing the expression of the *GADD153* gene.

There is little research on how the two isoforms of CRP interact with the apoptosis process. It is suggested that CRP can exert anti-apoptotic activity but only when the cyclic pentameric structure is lost. This would suggest that the apoptotic activity of CRP is induced through the native isoform. Native CRP (nCRP) can bind to low-affinity IgG FcγRIIa (CD32) and IgG FcγRI (CD64), leading to depressed functional activities, degranulation, and the generation of superoxide by inducible respiratory burst. On the other hand, mCRP binds to low-affinity IgG FcγRIIIb (CD16) that can delay apoptosis by triggering the cell survival pathway in neutrophils, even at low concentrations (89).

The nCRP isoform has the ability to opsonize apoptotic cells and induce the phagocytosis of damaged cells. Removal of nCRP-bound apoptotic monocytes and macrophages may be *via* FcγR-mediated phagocytosis (84). CRP binds to apoptotic cells, inhibits the assembly of terminal complement components, and promotes the opsonization of apoptotic cells (89, 90).

## CRP and Nitric Oxide (NO)

C-reactive protein has the ability to attenuate NO production with a marked reduction in *in vitro* angiogenesis, cell migration, and capillary-like tube formation by CRP at concentrations known to cause cardiovascular risk (91). Eisenhardt et al. (15) showed that CRP upregulated the expression of adhesion molecules and inhibited endothelial nitric oxide synthase (eNOS) expression, indicating a role for CRP in the production of NO. Several studies have revealed that CRP inhibits NO production *via* downregulation of eNOS in cardiovascular endothelial cells, thereby inhibiting angiogenesis *in vitro* and promoting the pathogenesis of atherosclerotic vascular disease through vasoconstriction, leukocyte adherence, and inflammation (14, 91–93). Another study found that it was in fact the nCRP isoform that downregulated eNOS and thus impaired endothelial function in ApoE knockout mice, *via* a mechanism thought to involve iNOS (94). Eisenhardt et al. (15) provided evidence that nCRP suppresses endothelium-dependent NO-mediated dilation by activating the p38 mitogen-activated protein kinase (MAP kinase) pathway and NADPH oxidase, suggesting that multiple pathways could be interacting with this process.

In contrast, mCRP has the opposite effect, enhancing NO production in neutrophils *via* upregulation of eNOS (95) with reverse transcription polymerase chain reaction showing an amplification of eNOS mRNA, but not iNOS or nNOS mRNA. This study highlighted that mCRP initiates calcium (Ca^2+^) mobilization and activation of calmodulin and PI3 kinase to induce NO formation in neutrophils (95). The effect of CRP isoforms on other inflammatory cells, such as monocytes or macrophages, has not been investigated to date.

## CRP Isoforms and Inflammatory Cytokines

There has been increasing evidence of a relationship between CRP and several pro-inflammatory cytokines.

### IL-6 and CRP

Interleukin-6 is a pro-inflammatory cytokine secreted by various cells including inflammatory cells, keratinocytes, fibroblasts, and endothelial cells. It regulates the acute-phase response, and its main role involves the host response to infection (96). Even though it is predominantly a pro-inflammatory cytokine, in some cells, IL-6 can have regenerative and anti-inflammatory effects through the activation of membrane-bound IL-6 receptor signaling (97).

Interleukin-6 is synthesized in the initial stages of inflammation and induces a number of acute-phase proteins, including CRP (98). IL-6 can also reduce the production of fibronectin, albumin, and transferrin as well as the promotion of CD4^+^ T helper cells, which initiates the linking of innate and acquired immunity (98). There is a correlation between increasing levels of IL-6 during inflammation and increasing levels of CRP (11), with IL-6 inducing the *CRP* gene (12). However, most investigations of CRP production by IL-6 generally fail to indicate which isoforms of CRP are generated. In some cases, the antibodies used suggest that nCRP is present, but given IL-6 occurs at the sites of inflammation, the pentameric CRP may be dissociating into mCRP.

When CRP levels become elevated in atheroma, this leads to the induction of IL-6 by macrophages indicating that CRP may have a direct effect on IL-6 release (99). Krayem et al. (100) found that a combination of mCRP, nCRP, and oxLDL decreases IL-6 production in a model of atherosclerosis. This triple combination suggests that nCRP might downregulate the IL-6 release by macrophages that have been stimulated by both mCRP and oxLDL.

### Interleukin-8 (IL-8) and CRP

Interleukin-8 is a cytokine produced by numerous cell types including inflammatory cells, keratinocytes, fibroblasts, and endothelial cells. IL-8 acts as a potent chemoattractant of neutrophils (101) and is overexpressed in chronic inflammatory diseases and during septic shock (102). IL-8 stimulates the release of granules from neutrophils by a process called degranulation. These granules contain a range of antimicrobial effectors that can help combat infection (103). Neutrophils are the first inflammatory cells to arrive at the site of inflammation, and they carry out the phagocytosis of bacteria and release chemotactic mediators that recruit other leukocytes to the affected tissue (103).

Kibayashi et al. (104) indicated that CRP plays a role in atherosclerosis *via* enhanced IL-8 production and increased expression of IL-8 mRNA in a CRP dose-dependent manner. They showed that CRP promotes IL-8 production *via* the activation of the ERK, p38 MAPK, and JNK pathways. Conversely, Wigmore et al. (105) indicated that IL-8 induces CRP production in hepatocytes, providing a potential feedback loop. The effect of the different CRP isoforms on IL-8 production has been investigated. Khreiss et al. (37) showed that nCRP had no detectable effect on the production of IL-8, whereas mCRP increased IL-8 production and IL-8 gene expression, promoting pro-inflammatory activity through a p38 MAPK-dependent mechanism. When treated with anti-CD16, there was inhibition of mCRP-stimulated NO formation and IL-8 release.

### MCP-1 and CRP

Monocyte chemoattractant protein-1 is a cytokine that plays a role in the regulation of migration and infiltration of monocytes and macrophages (106). It is released by a number of cell types in response to events such as oxidative stress, cytokine release, and growth factor release (107). Human MCP-1 is known to bind to at least two receptors, and its production can be induced by interleukin-4 (IL-4), IL-1, TNF-α, bacterial LPS, and IFN-γ (107). There is increasing evidence that MCP-1 influences T-cell immunity by enhancing the secretion of IL-4 by T cells, as well as having a role in the migration of leukocytes (106). This in turn has a regulatory function on monocytes and macrophages, which are the major source of MCP-1 (107). MCP-1 is known to recruit monocytes to the vessel wall (99) and cause the arrest of rolling monocytes on endothelial monolayers that express E-selectin (108).

Evidence suggests that CRP stimulates endothelial cells to express MCP-1 (99) in addition to being a direct chemoattractant of monocytes itself (109). CRP can promote monocyte chemotactic activity in response to MCP-1 *via* upregulation of the monocyte chemotaxis receptor CCR2, with elevated CRP levels promoting the accumulation of monocytes in the atherogenic arterial wall (99). When vascular smooth muscle cells are exposed to increasing levels of CRP, MCP-1 mRNA substantially increased within 2 h and remained elevated for at least 24 h (110). Incubation with mCRP increases the secretion of MCP-1, leading to pro-inflammatory activity through a p38 MAPK-dependent mechanism, whereas nCRP had no detectable effect (37).

### TNF-α and CRP

Tumor necrosis factor-α is a component of the acute-phase response and is mainly produced by monocytes and macrophages but can be produced by numerous other immune cells such as neutrophils, natural killer cells, and eosinophils. TNF-α is not usually detectable in a healthy host, but levels become elevated in a number of inflammatory and infectious conditions (111). The main stimulant of TNF-α production is LPS, but many other pathological conditions such as trauma infection, impaired wound healing, and heart failure also induce its production (111, 112). TNF-α mediates various processes such as cell proliferation, differentiation, and apoptosis.

Studies have shown a correlation between TNF-α production and the concentration of CRP. TNF-α induces a dose-dependent secretion of CRP in hepatocytes, which corresponds to an increase in CRP mRNA (28). Conversely, elevated CRP levels in atheroma leads to the induction of IL-1β, IL-6, and TNF-α production by macrophages (99). Research shows a close relationship between TNF-α and IL-6 levels in inflammation (113), with both TNF-α and IL-6 inducing the transcription of CRP (33). However, there is some contradictory evidence showing a potential inhibitory effect of CRP on TNF-α production, suggesting that there could be a negative feedback mechanism whereby elevated levels of CRP inhibit further stimulation of CRP by reducing the TNF-α production (114). A combination of mCRP, nCRP, and oxLDL also causes a decrease in both TNF-α and IL-6 production in a macrophage model of atherosclerosis (100). This triple combination suggests that nCRP might downregulate TNF-α and IL-6 production by macrophages stimulated by both mCRP and oxLDL.

## Conclusion

C-reactive protein is a homopentameric acute-phase inflammatory protein that exhibits elevated expression during inflammatory conditions such as rheumatoid arthritis, some cardiovascular diseases, and infection. Evidence suggests that CRP is an important regulator of inflammatory processes and not just a marker of inflammation or infection. Key areas of inflammation and host responses to infection mediated by CRP include the complement pathway, apoptosis, phagocytosis, NO release, and cytokine production. However, most research to date has investigated the role of CRP in the vascular tissues, highlighting the need to conduct further work to determine the precise role of CRP in peripheral tissues.

C-reactive protein is synthesized primarily in liver hepatocytes but also other cell types such as smooth muscle cells, macrophages, endothelial cells, lymphocytes, and adipocytes. Evidence also suggests that the sex steroid hormone estrogen can influence CRP levels, with HRT having a profound influence on CRP levels in the elderly. Administration of oral HRT increases background levels of CRP in circulation, whereas evidence suggests that transdermal estrogen supplementation either reduces or has little effect on circulating CRP levels. A reduction in CRP levels following local administration of estrogen supports findings showing that estrogen reduces the inflammatory response in peripheral tissues such as skin.

There are two distinct isoforms of CRP, nCRP and mCRP, and the nCRP isoform can irreversibly dissociate at sites of inflammation, tissue damage, and infection into five mCRP subunits. Evidence indicates that nCRP often tends to exhibit more anti-inflammatory activities compared to mCRP. The nCRP isoform activates the classical complement pathway, induces phagocytosis, and promotes apoptosis. On the other hand, mCRP promotes the chemotaxis and recruitment of circulating leukocytes to areas of inflammation and can delay apoptosis. The nCRP and mCRP isoforms inhibit and induce NO production *via* downregulation and upregulation of eNOS, respectively. In terms of pro-inflammatory cytokine production, mCRP increases IL-8 and MCP-1 production, whereas nCRP has no detectable effect on their levels. CRP can also induce IL-6 and TNF-α production at sites of inflammation, again suggesting probable involvement of mCRP from the dissociation of nCRP. Further studies are needed to expand on these emerging findings and to fully characterize the differential roles that each CRP isoform play at sites of local inflammation and infection.

## Author Contributions

Both authors contributed equally to the planning, preparation, drafting and writing of the article.

## Conflict of Interest Statement

The authors declare that the research was conducted in the absence of any commercial or financial relationships that could be construed as a potential conflict of interest. The handling Editor declared a shared affiliation, though no other collaboration, with the authors.
